# Hepatitis C Virus Infection, Linxian, China^1^

**DOI:** 10.3201/eid1101.031005

**Published:** 2005-01

**Authors:** Mingdong Zhang, Xiu-Di Sun, Steven D. Mark, Wen Chen, Lara Wong, Sanford M. Dawsey, You-Lin Qiao, Joseph F. Fraumeni, Philip R. Taylor, Thomas R. O’Brien

**Affiliations:** * National Institutes of Health, Bethesda, Maryland, USA; † Cancer Institute, Chinese Academy of Medical Sciences, Beijing, China

**Keywords:** AIDS, China, epidemiology, hepatitis B virus (HBV), hepatitis C virus (HCV), human immunodeficiency virus type 1 (HIV-1), liver cancer, prevalence, research

## Abstract

Bloodborne viruses may have spread in rural China during the past 25 years, but population-based prevalence estimates are lacking. We examined the frequency of hepatitis C virus (HCV) and HIV type 1 (HIV-1) among residents of Linxian, a rural community in Henan Province. In 2000, blood was collected from participants (>55 years of age) who had enrolled in a population-based nutritional intervention trial in 1985. We randomly selected 500 participants for HCV testing and 200 participants for HIV-1 testing. For HCV, 48 (9.6%) of 500 participants were positive by enzyme immunoassay and recombinant immunoblot assay (95% confidence interval [CI], 7.0%–12.2%), and prevalence was lowest in the most geographically isolated participants. Among the HCV-infected participants, 42 had a specimen available from1985, of which 16 (38.1%) were positive for HCV. For HIV-1, 0/200 participants were positive. We conclude that HCV is now a common infection among older adults in Linxian, China.

## Abstract 2

The prevalence of HCV infection was high among older citizens of Linxian, China, in 2000.

Hepatitis C virus (HCV) infection is becoming a global public health problem (*1*). The overall prevalence of HCV infection is 1%–2% in most countries that have been studied (*2*), but the distribution of HCV varies considerably among populations. HCV is most frequently transmitted by percutaneous exposure to infectious blood or blood-derived body fluids, such as through transfusion of contaminated blood or blood products, nonsterile medical injections, or injection drug use. Very high rates of HCV infection are found among persons exposed to HCV through these routes (*3*). In a nationwide study conducted in 1992, HCV prevalence was reported to be 3.2% in China overall and 3.1% in rural China (*4*). Other smaller studies have reported HCV prevalence rates of 0% to 3% in rural populations from various Chinese provinces (*5*–*7*).

HIV type 1 (HIV-1) infection has been reported among paid blood donors in rural east central China (*8*–*10*). Because both HIV-1 and HCV are bloodborne, these reports raise the possibility that HCV may be common in this area. To date, population-based rates of bloodborne viral infections in this region are lacking. To provide such estimates, we used specimens collected as part of a population-based study in Linxian, Henan Province, a rural county in central China where farming is the predominant occupation (Figure).

**Figure:**
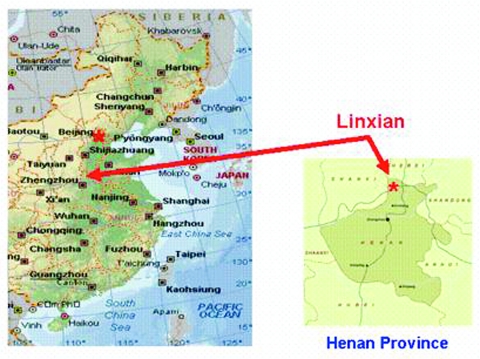
Eastern part of China showing the locations of Linxian and Henan Provinces. Adapted from www.Expedia.com.

In 2000, surviving participants of a population-based nutritional intervention trial were resurveyed. The study had been initiated to study the effect of dietary supplements on the risk of esophageal and gastric cancer, which occur at very high rates in this region. Participants were healthy adults from 4 Linxian communes who were 40–69 years of age when enrolled in 1985. We used specimens collected in this study to estimate the prevalence of hepatitis B virus (HBV), HCV, and HIV-1 among older adults in Linxian in 2000.

## Methods

### Participants and Samples

In 1985, all healthy adults aged 40-69 years from 4 Linxian communes (Yaocun, Rencun, Donggang, and Hengshui) were invited to participate in the Linxian Nutritional Intervention Trial (NIT). NIT is a population-based trial designed to prevent esophageal and gastric cancer, the most common cancers in the Linxian region. Of ≈53,000 potentially eligible participants, 16% refused participation, 12% were not living in Linxian at the time due to temporary employment, 4% were excluded for medical reasons, and 8% did not join the trial for other reasons (*11*). The remaining 32,902 members of the Linxian population participated in the trial. In 1985, 1 year before the start of the intervention, each participant was interviewed, was given a brief physical examination, and had blood drawn.

In 2000, all 23,910 surviving participants were invited to take part in a follow-up survey; 16,488 participants were then resurveyed and had blood samples collected. Analysis of the cause of death from 1985 to 2001 indicated that most deaths were due to esophageal or gastric cancer (28%), heart disease (21%), or stroke (31%). About 2% of deaths were due to cirrhosis and another 2% were due to liver cancer. Higher death rates among men and older participants shifted the demographic pattern among survivors compared to original enrollees. For example, men comprised 40% of participants in 2000 and 44% in 1985). No difference in the distribution of commune of residence was found between survivors and all enrollees.

Ten milliliters of blood was collected in vacutainers that contained sodium heparin; the samples were stored at room temperature and transferred within 3 hours to a field station for processing. Aliquots of plasma were stored at –85°C until they were shipped on dry ice to a biospecimen repository in Frederick, Maryland. Participants in the 2000 blood collection survey received the results of their blood samples, including hematocrit and liver function tests. The study protocol was approved by Institutional Review Boards at the U.S. National Cancer Institute and the Cancer Institute of the Chinese Academy of Medical Sciences.

For HBV and HCV testing, we selected at random 500 NIT participants who were resurveyed in 2000. In addition, we tested available specimens that had been drawn in 1985 among participants who we found to be positive for anti-HCV by recombinant immunoblot assay (RIBA) in 2000. We also randomly selected another 200 NIT participants from participants resurveyed in 2000 for HIV-1 antibody testing. Because HIV-1 testing was not within the scope of the original study, we removed identifying information from these 200 samples and tested them anonymously.

### Laboratory Assays

We tested the plasma specimens for HCV antibody by HCV Version 3.0 enzyme immunoassay (EIA) (Ortho Diagnostics, Raritan, NJ) according to the manufacturer’s instructions. Samples that were positive by HCV enzyme immunoassay EIA were confirmed by HCV Version 3.0 RIBA (Ortho Diagnostics). Because the specimens had been collected in sodium heparin, which inhibits the polymerase chain reaction (PCR), we could not confirm the presence of HCV by PCR-based assays. As an alternative, we tested specimens for HCV core antigen (Trak-C, Ortho Diagnostics). HCV core antigen is a marker of chronic infection and the analytic sensitivity of the Trak C assay (Ortho Diagnostics) is similar to that of PCR-based assays for HCV RNA (*12*). The 500 samples were also tested for antibody to HBV core antigen (anti-HBc) by an HBc ELISA (Ortho Diagnostics). Samples that were positive for anti-HBc were tested for HBV surface antigen (HBsAg) by EIA (Bio-Rad, Redmond, WA). A separate group of 200 samples was tested for HIV-1 antibody by EIA (Genetic Systems rLAV EIA, Bio-Rad).

### Statistical Methods

Chi-square analysis was used to compare the prevalence of viral infections among participants with different demographic characteristics. The 95% confidence interval (95% CI) of prevalence was calculated based on normal approximation to binomial distribution when that approximation holds. For rare events, the 95% CI was based on the Poisson approximation to the binomial distribution. All statistical analyses were done with the Statistical Analysis System version 8.0 (Cary, NC).

## Results

Demographic characteristics of the 500 participants tested for HCV are summarized in the Table. In 2000, more than half of the participants were 55–64 years of age (54.2%), 34.4% were 65–74 years, and 11.4% were 75–84 years. This age distribution reflects that participants were aged 40–69 years when enrolled in 1985. Our study included 200 (40.0%) men and 300 (60.0%) women. One hundred seventy (34.0%) participants were from Yaocun, 95 (19.0%) from Rencun, 116 (23.2%) from Donggang, and 119 (23.8%) from Hengshui.

Of the 500 participants studied, 63 (12.6%) were positive for HCV antibody by EIA. When the specimens from these 63 participants were tested by HCV RIBA, 48 were positive, 7 were indeterminate, and 8 were negative. Therefore, the estimated prevalence of HCV infection (defined as positive by both HCV EIA and HCV RIBA) in this population in 2000 was 9.6% (95% CI 7.0%–12.2%). We tested all 23 samples that were positive for HCV RIBA and had sufficient remaining plasma for HCV core antigen. HCV core antigen was found in 16 (69.6%) of 23 specimens.

The prevalence of HCV infection did not differ meaningfully by age or sex (Table), but varied significantly among the four communes. Donggang, the most geographically isolated commune, had a lower prevalence of HCV infection (2.6%) than Yaocun (11.8%, p < 0.01), Rencun (14.7%, p < 0.01), or Hengshui (9.2%, p = 0.03; p = 0.02, overall chi-square test). No other significant differences in HCV prevalence were found between the communes.

**Table:** Prevalence of anti-HCV (by RIBA), HBsAg, and HBcAb in Linxian in 2000*

	N (%)	Anti-HCV % (95% CI)	p	HBcAb % (95% CI)	p	HBcAb and HBsAg % (95% CI)	p
Overall		9.6 (7.0–12.2)		54.6 (50.2-59.0)		6.4 (4.3-8.5)	
Age in 2000 (y)							
<59	166 (33.2)	8.4 (4.2–12.7)		53.6 (46.0–61.2)		5.4 (2.0–8.9)	
60–64	105 (21.0)	13.3 (6.8–19.8)		50.5 (40.9–60.0)		6.7 (1.9–11.4)	
65–69	90 (18.0)	11.1 (4.6–17.6)		50.0 (39.7–60.3)		4.4 (0.2–8.7)	
70–74	82 (16.4)	6.1 (0.9–11.3)		62.2 (51.7–72.7)		8.5 (2.5–14.6)	
>75	57 (11.4)	8.8 (1.4–16.1)	0.50	61.4 (48.8–74.0)	0.34	8.8 (1.4–16.1)	0.73
Sex							
Male	200 (40.0)	9.5 (5.4–13.6)		58.0 (51.2–64.8)		5.0 (2.0–8.0)	
Female	300 (60.0)	9.7 (6.3–13.0)	0.95	52.3 (46.7–58.0)	0.21	7.3 (4.4–10.3)	0.18
Commune							
Yaocun	170 (34.0)	11.8 (6.9–16.6)		53.5 (46.0–61.0)		6.5 (2.8–10.2)	
Rencun	95 (19.0)	14.7 (7.6–21.9)		64.2 (54.6–73.8)		6.3 (1.4–11.2)	
Donggang	116 (23.2)	2.6 (0.0–5.5)		53.5 (44.4–62.5)		6.0 (1.7–10.4)	
Hengshui	119 (23.8)	9.2 (4.0–14.4)	0.02	49.6 (40.6–58.6)	0.18	6.7 (2.2–11.2)	0.50

*HCV, hepatitis C virus; RIBA, recombinant immunoblot assay; HBsAg, hepatitis B surface antigen; HBcAb, hepatitis B core antibody; CI, confidence interval.

Among the 48 participants who were positive by HCV RIBA in 2000, 42 had specimens available from 1985 for HCV antibody testing. Of these 42, 16 (38.1%) were positive by both HCV EIA and HCV RIBA, indicating that most participants who were infected with HCV in 2000 had acquired the virus since 1985.

Antibodies to HBV core antigen (HBc), an indicator of past exposure to HBV, were found in 273 (54.6%) of 500 participants. The prevalence of anti-HBc did not vary significantly by age, sex, or commune (Table). Participants who were infected with HCV had a slightly higher prevalence of anti-HBc than those who were not (64.6% vs. 53.5%, p =0.14). Participants who were positive for both anti-HBc and hepatis B surface antigen (HBsAg), a marker for chronic HBV infection, comprised 6.4% of the population. The prevalence of HBsAg differed relatively little by age, sex, or commune (Table).

A separate group of 200 participants was randomly selected for anonymous testing for antibodies to HIV-1. These participants included 77 men (38.5%) and 123 women (61.5%). Age as of the year 2000 was distributed as follows: <59 years, 78 (39.0%); 60–69 years, 76 (38.0%); >70 years, 46 (23.0%). Seventy-two participants (36.0%) were from Yaocun, 29 (14.5%) were from Rencun, 48 (24.0%) were from Donggang, and 51 (25.5%) were Hengshui. None tested positive for antibodies to HIV-1. The 95% CI for HIV-1 prevalence in this population was 0%–0.03%.

## Discussion

In this population-based study of 55- to 84-year-old persons living in a rural area of Henan Province, HCV prevalence in 2000 was 9.6%. HCV was present in the Linxian population in 1985, but most of the HCV-infected participants in this study likely acquired the virus between 1985 and 2000. In contrast to the high rate of HCV infection, HIV-1, which also is transmitted through infected blood, was not found in this population. Evidence of resolved or chronic HBV infection, which is endemic in China, was found in about 55% of the participants.

The observed prevalence of HCV in Linxian is higher than in most previous population-based studies from China. In the nationwide cross-sectional study conducted in 1992, HCV prevalence was 3.10% in residents of rural China and 3.96% in the group aged 50 to 59 years, the eldest in that study (*4*). However, because HCV prevalence in the nationwide study was determined by HCV EIA alone, estimates from that study are higher than would have been obtained with confirmation by HCV RIBA. The RIBA-confirmed HCV prevalence in Linxian was, therefore, considerably higher than the national rate.

Around the world, the prevalence of HCV infection appears to be low in most populations (*13*–*15*), but areas of high prevalence have been found. Perhaps the most notable example is Egypt, where >15% of the population may be infected with HCV (*16*,*17*). Transmission of HCV occurred in Egypt from the 1960s through the 1980s when a campaign against schistosomiasis involved mass parenteral injections, and unsterilized needles were used (*17*). Evidence also exists that HCV infection may have been transmitted in Egypt through other types of medical care (*18*). Iatrogenic transmission may have contributed as well to high rates of HCV infection that have been reported from Taiwan (*19*), Japan (*20*), and Italy (*21*).

We could not determine how HCV spread in Linxian because the nutritional trial did not collect information on potential exposure to contaminated blood. Widespread HCV infection in a population generally results from iatrogenic transmission or sharing of recreational drug injection equipment. HCV spreads rapidly among injection drug users, but, to our knowledge, reports of injection drug use in China are limited to younger age groups in border provinces and large cities (*22*,*23*), not older residents of inland provinces. It seems unlikely, therefore, that our participants acquired HCV infection through injection drug use. Transmission of HCV has been linked to paid blood and plasma donations in Central Chinese provinces, including Henan, during the 1980s and 1990s (*24*,*25*). Reuse of needles and equipment without proper sterilization and reinfusion of pooled red blood cells from multiple donors reportedly led to outbreaks of HCV (*24*,*25*). These reports, along with evidence from our study that many HCV-infected participants acquired the virus between 1985 and 2000, suggest that HCV may have been transmitted among the Linxian population through blood or plasma collection activities. Our finding of the lowest HCV prevalence (2.6%) in the most isolated commune seems consistent with this explanation, since geographic isolation may have limited the opportunity to contract HCV infection through these means.

Outbreaks of HIV-1 have been reported among paid plasma donors in central Chinese provinces, including Henan (*8*–*10*), but we found no evidence of HIV-1 infection among 200 randomly selected participants. Our analysis yielded an upper 95% CI of 0.03% for the prevalence of HIV-1 in this population, which indicates that HIV-1 infection is, at worst, extremely rare among older residents of Linxian. The absence of HIV-1 in the presence of a relatively high prevalence of HCV is not surprising because the entry of either virus into a community may depend to some degree on chance.

HBV infection is endemic in China (*26*), and most transmission occurs during the perinatal period when the risk of chronic infection is much higher than in adulthood. Among our participants, 54.6% had HBc, and 6.4% had both anti-HBc and HBsAg (which indicates chronic infection). Most or all of the participants who were chronically infected with HBV likely became infected early in life. More recent bloodborne transmission may have contributed some participants with resolved HBV infection, as suggested by the trend toward higher anti-HBc prevalence among participants who were also infected with HCV.

The accuracy with which our estimates reflect the prevalence of viral infections among older Linxian residents in the year 2000 depends on 2 factors: the test characteristics of the assays we used and how well our study population represents the target population. The third generation HCV EIA we used has high sensitivity and specificity. With confirmation of HCV EIA-positive samples by a highly specific RIBA, we are unlikely to have overestimated the prevalence of HCV antibodies among our participants. We have similar confidence in the assays that we used for HIV-1 and HBV testing.

For practical reasons, we used specimens that had been collected as part of a population-based research effort that began in 1985. The limitations of our approach for determining the prevalence of viral infections among Linxian residents in 2000 should be considered. First, the age criterion of the original study prevented us from ascertaining infection prevalence among persons <55 years of age. HCV prevalence may be higher or lower among younger residents of Linxian. Second, the participants may not represent older adults residing in Linxian in 2000 with regard to viral prevalence. About 60% of eligible residents enrolled in the trial in 1985 and about 70% of surviving enrollees participated in the 2000 follow-up. If surviving enrollees who were infected with one of the viruses that we studied were less likely to participate in the 2000 survey due to poor health, we may have underestimated the true prevalence of infection. However, usually a long period elapses from infection with these viruses to disease, and our results likely provide a reasonable estimate of viral prevalence among older residents of Linxian.

In summary, HCV is now common in this rural Chinese community, at least among its older residents. In contrast, we found no evidence of HIV-1 infection in the population. The public health impact of the high prevalence of HCV infection in Linxian may be substantial. HCV is an important cause of end-stage liver disease and hepatocellular carcinoma, and it can act synergistically with HBV infection (*27*), which is endemic in China. Future studies should examine the prevalence of bloodborne viruses in other parts of China, how these viruses are transmitted, and the resulting health effects. Efforts to halt the transmission of HCV and other bloodborne viruses in rural China should be a top public health priority.
